# Supplementary figures and images for: Extraction, analysis, and antifungal activity study of algae antibiotic active substances in plateau lakes
Source: PLoS One. 2025 May 8;20(5):e0319853. doi: 10.1371/journal.pone.0319853 (PMC12061390; doi:10.1371/journal.pone.0319853)

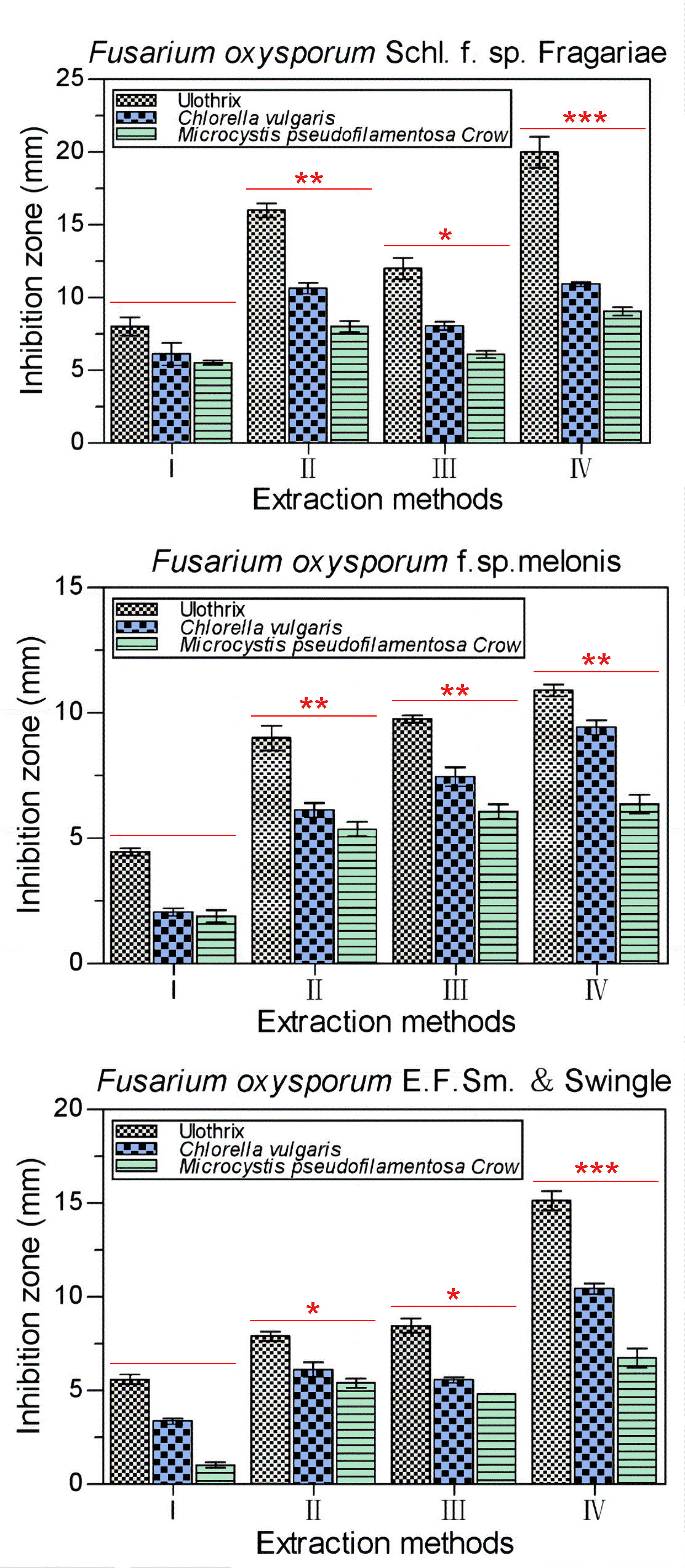

Supplement: S1 Fig — Note: The vertical axis is inhibition zone(mm), and the horizontal axis is extraction menthods; I: Improved lime sulfur method; II: Ethyl acetate extraction method; III: Ethanol reflux extraction method IV: Methanol extraction method. Different colors represent different algae. Data are expressed as means ± SD of four separate experiments. *P < 0.05, **P < 0.01, ***P < 0.001, when compared to the control. (TIF) [file pone.0319853.s001.tif]

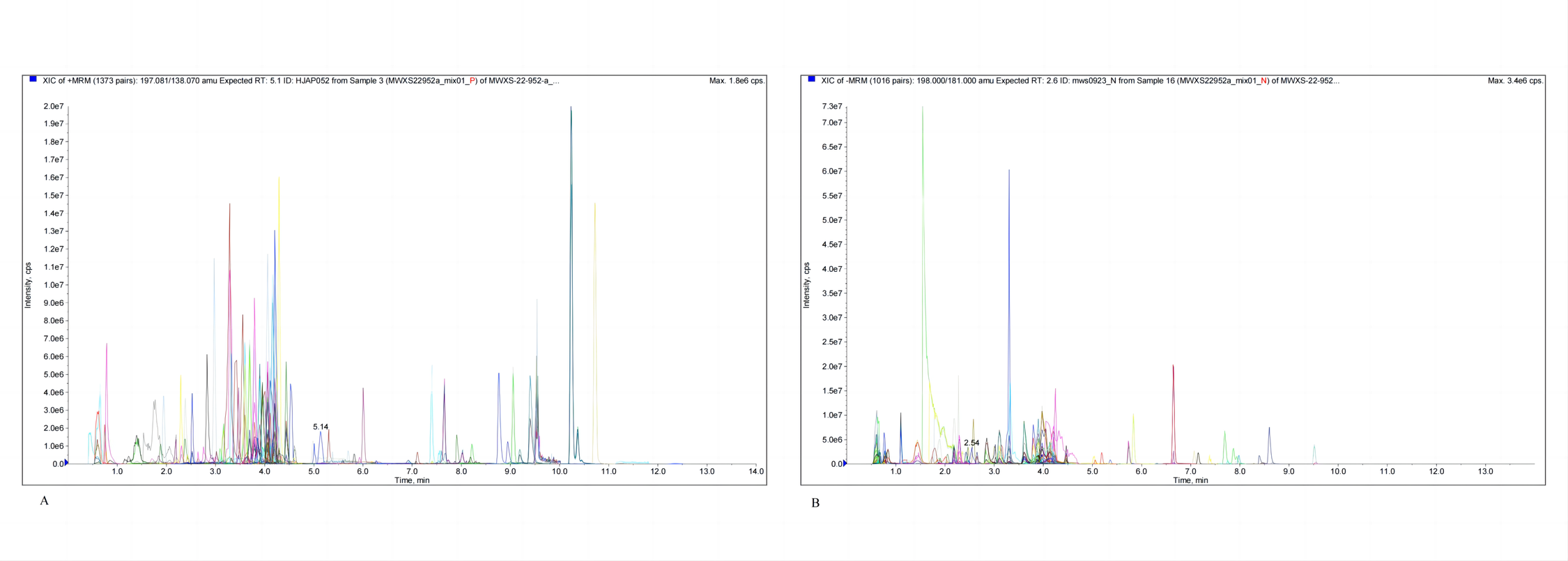

Supplement: S2 Fig — Note: A. Mixed sample of methanol extracts of the algae (positive ion mode), and B. Mixed sample of methanol extracts of the algae (negative ion mode). (TIF) [file pone.0319853.s002.tif]

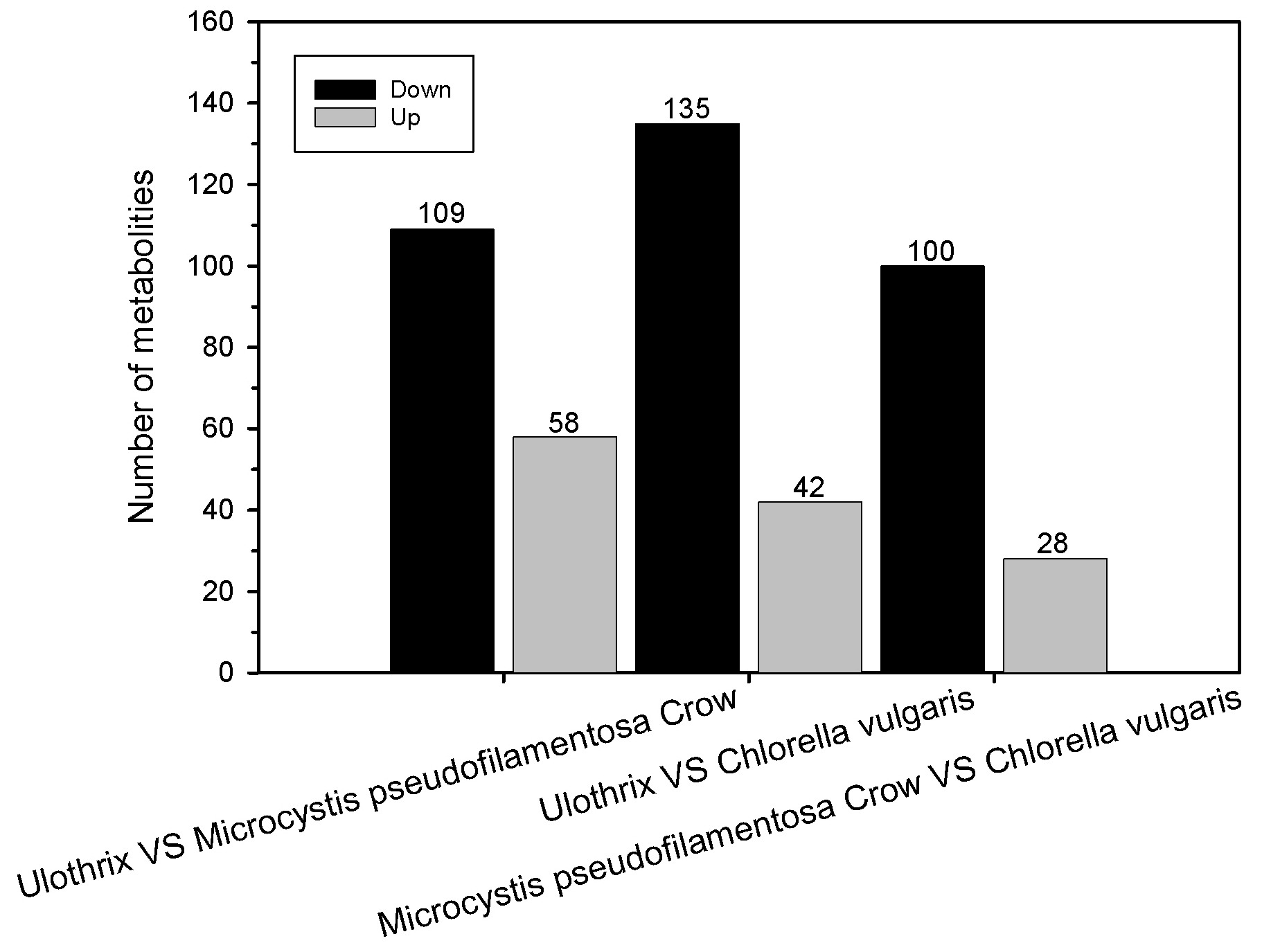

Supplement: S3 Fig — Number of differentially metabolities identified in three comparision groups. (TIF) [file pone.0319853.s003.tif]

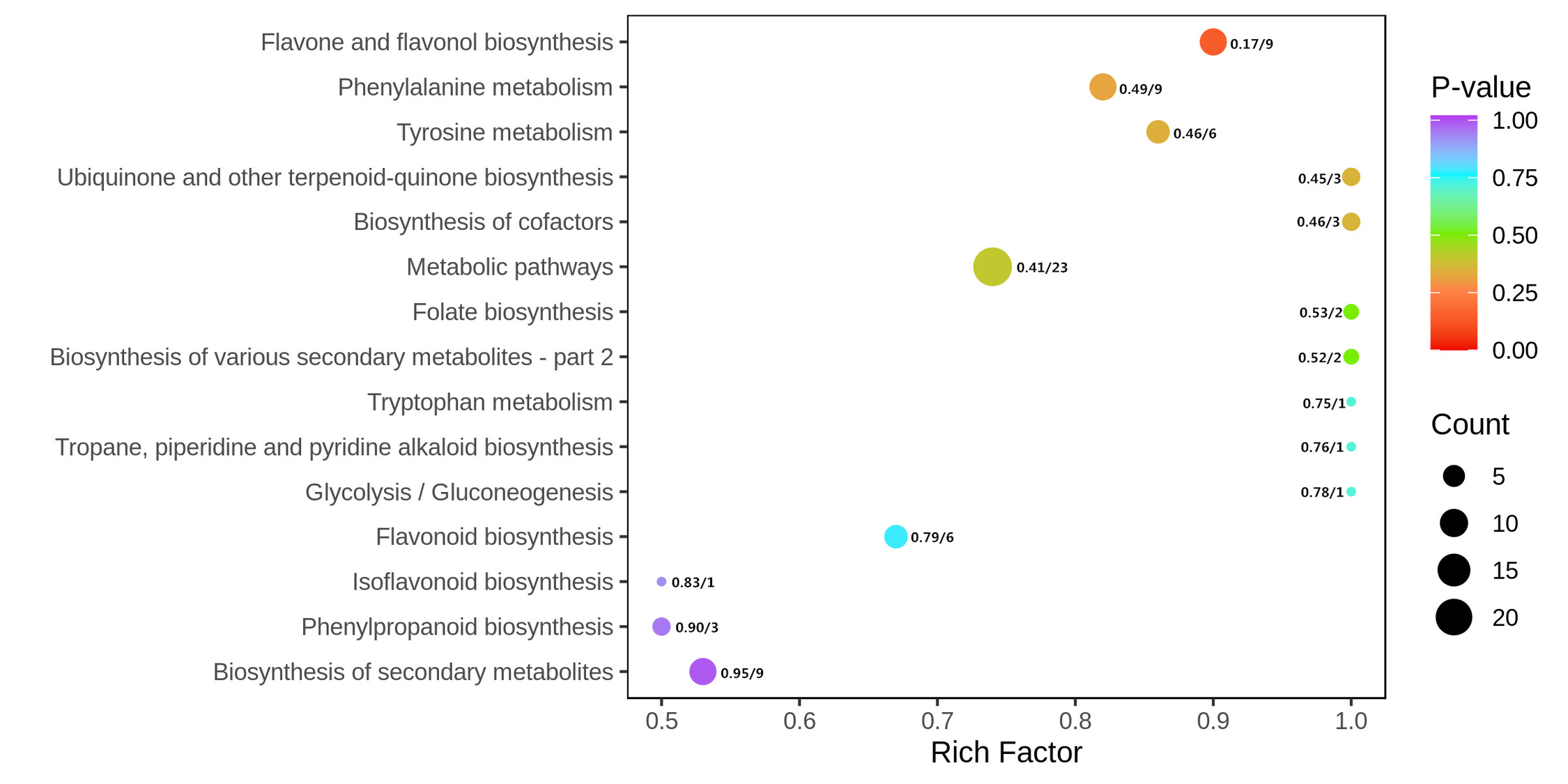

Supplement: S4 Fig — Note: The abscissa represents the rich factor corresponding to each pathway. The ordinate represents the pathway name (sorted according to P-value). The color of the point represents the P-value size, with greater color intensity indicating a more significant enrichment. The size of the dots represents the number of enriched differential metabolites, the larger the dot, the greater the number of metabolites(P-value/Count). (TIF) [file pone.0319853.s004.tif]
