# Supplementary material for: Extraction, analysis, and antifungal activity study of algae antibiotic active substances in plateau lakes
Source: PLoS One. 2025 May 8;20(5):e0319853. doi: 10.1371/journal.pone.0319853 (PMC12061390; doi:10.1371/journal.pone.0319853)
Supplement: Attachment — Submitted filename: Response to reviewers.docx [file pone.0319853.s007.docx]

Dear Editor-in-Chief

We would like to have our revised version of manuscript entitled “**Extraction, analysis, and antifungal activity study of algae antibiotic active substances in plateau lakes”** considered for publication in **“Plos one”.**

All the comments and suggestions are addressed in the revised version and track

changes are mentioned inside the revised file for editors and reviewers.

**Extraction, analysis, and antifungal activity study of algae antibiotic active substances in plateau lakes**

**Reviewer 1:**

**Question1**: Introduction: The study background and goals could be explained more clearly in the opening. It would be helpful to make it clear what the study's main goal is and why it's important to look into polyphenol molecules from algae in the context of fighting fungus pathogens.

**Author Response:** Many thanks for this comment. We have supplemented the application of biogenic phenolic acid compounds in the prevention and control of plant fungal diseases. In addition, we also presented the significance of studying phenolic acid compounds in algae.

In order to make it more clear to the readers, the explanation is added in the introduction part line 64-73,page 2-3.

**Question2**: Methodology: While the methods section provides a detailed description of the extraction procedures, it would benefit from additional details regarding the validation of the analytical techniques employed for assessing polyphenol content and metabolite identification. Including validation parameters like linearity, accuracy, precision, and limits of detection and quantification would enhance the credibility of the findings.

**Author Response:** We provide validation parameters for measuring linearity, precision, stability, etc.Line135-142, page5.

**Question3**:Results: The results section contains a variety of information, including polyphenol content, antifungal activity, and metabolite characterization. To increase clarity, divide the findings into subsections according to each target (for example, Extraction Efficiency, Antifungal Activity, Metabolite Identification). This would make it easier for readers to understand the results. Also when presenting numerical data, consider providing measures of variability (e.g., standard deviation) along with the mean values.

**Author Response:**Thanks for this important suggestion. We have adjusted the structure of the results section.

**Question4**:Discussion: The discussion effectively explains how the results align with the study's goals. Yet, it could be improved by linking the findings to previous research. This means comparing them with past studies on polyphenols from algae and their antifungal effects. Doing this would better explain the significance of the results.

**Author Response:** We have supplemented and improved the discussion section to provide readers with a clearer understanding of the significance and highlights of this study.

**Question5**: Conclusion: While the conclusion provides a concise summary of the study's key results, it might be enhanced to provide more specific implications for further research or real-world situations. In particular, a discussion of the importance of the discovered metabolites and how they may be used as organic fungicides in farming would improve the end section

**Author Response:** Thanks. We have supplemented and elaborated on the application prospects of algal phenolic acid metabolites in the prevention and control of agricultural diseases. Line 261-272, page 11.

**Question6**: Language and Clarity: Pay attention to sentence structure and clarity throughout the manuscript. Some sentences are long and complex, which could make it difficult for readers to follow the logic. Simplifying the language and breaking down complex ideas into shorter sentences would improve readability.

**Author Response:** We have made adjustments to some statements to improve readability.

**Reviewer 2:**

Experiment have been conducted rigorously with appropriates contrals and replication. Sample sizes was enough to produce robust results. Methods have been described in sufficient detail. The data presented in the munuscript support the concludion drawn. the article have been weitten in standard English and easy to understan.

**Author Response:** Thank you for recognizing our research findings
