# Supplementary material for: Extraction, analysis, and antifungal activity study of algae antibiotic active substances in plateau lakes
Source: PLoS One. 2025 May 8;20(5):e0319853. doi: 10.1371/journal.pone.0319853 (PMC12061390; doi:10.1371/journal.pone.0319853)
Supplement: Attachment — Submitted filename: Response_to_reviewers_auresp_2.docx [file pone.0319853.s008.docx]

Dear Editor-in-Chief

We would like to have our revised version of manuscript entitled “**Extraction, analysis, and antifungal activity study of algae antibiotic active substances in plateau lakes”** considered for publication in **“Plos one”.**

All the comments and suggestions are addressed in the revised version and track

changes are mentioned inside the revised file for editors and reviewers. The raw data has been uploaded to the supporting Information.

**Extraction, analysis, and antifungal activity study of algae antibiotic active substances in plateau lakes**

**Reviewer 3:**

**Question1**: I have gone through the manuscript tittled 'Extraction, analysis, and antifungal activity study of algae antibiotic active substances in plateau lakes. A lot of information is missing or not made available. and yet these are your results. The conclusions are not supported by the available data forexample; From line 197-199, i.e.242 polyphenolic metabolites were preliminarily identified, of which 160 were phenolic acids. You must provide the chromatograms and a table of identified 242 metabolites and a write up on how you identified the metabolites. Otherwise the information provided does not support the conclusions.

**Author Response:** Many thanks for this comment. Due to the consideration of the large number of metabolites (242), it was inconvenient to list them in the article, so they have been uploaded as supporting data.

**Question2**: Your supplied chromatograms are not vissible or clear

**Author Response:** Thanks for this important suggestion. We have already updated the chromatograms.

**Question3**:Even the statistical analysis is not supported by the data e.g. how did the authors run the Principle component analysis?

**Author Response:**Thanks for this important suggestion. We have added the mathods of qualitative and quantitative analysis of metabolites, PCA, Differential metabolites selected and Statistical analyses.

**Reviewer 4:**

**Question1**:Abstract – The abstract should include why this study has been performed e.g. explain the purpose and significance of the study and findings in one sentence. Currently, it is only described what has been done but the ‘why’ is lacking. Also, the tense isn’t consistent throughout the abstract e.g. there are parts were present tense - and others were past tense is used. Lastly, the different categories of molecules identified is mentioned, however, there are some that are quite unspecific e.g ‘other flavonoids’. This should be clarified or included in the flavonoid category.

**Author Response:** Many thanks for this comment.We have made adjustments to the abstract section, supplemented and updated the corresponding content.Line 29-32, 43 and 47, page 1.

**Question2**:Introduction – I recommend that this is restructured so that it is easier for the reader to understand the purpose of this study. The explanation that has been added from line 64-73 is well written and explains the importance and use of antifungal compounds, however, lines 56-64 simply listing previous findings seem out of place – e.g. this could be mentioned as ‘previous findings or examples’ after explaining the importance. Further, citation style is inconsistent throughout this section and inconsistent with parts of the discussion (e.g. 192, 194). It would also improve the manuscript if the authors could mention why these three types of algae have been investigated – was this because these are most prevalent in the region or simply because these were most accessible, or do these three represent a diverse group of algae?

**Author Response:**Thanks. We have restructured and revised the introduction section.Line 62-83 and 105-107, page 2-4.

**Question3**:Methods – Overall the methods used are appropriate, however some clarification should be provided to allow other researchers to apply the techniques to their own projects.It is not sufficient to mention pH as approximates if this is not further specified – provide either a specific value of the pH or a range that is suitable – e.g. is pH of approximately 10 equal to the range of pH 9.5 – 10.5? Or is this closer to pH 10 +/- 0.1? PH approximates are given in line 104, 106, 123. Further clarification is needed for procedures that mention ‘several times’ (line 110) or ‘an appropriate amount’ (line 111, 124). How often is ‘several times’ and what is an ‘appropriate amount’?A data analysis section should be added. Currently, there is no explanation regarding what software was used to extract/collect the data and what was used for analysis. E.g. GraphPad Prism, Sample size (n number), what was considered significant etc. This is currently lacking in the result section and figures as well.

**Author Response:**Thanks for this important suggestion. We have added the mathods of qualitative and quantitative analysis of metabolites, PCA, Differential metabolites selected and Statistical analyses. In addition, we have clarified the specific pH value in the text and corrected the unclear expression. Line 62-83 and 122-152, page 5-6.

**Question4**:Conclusion – The conclusion is sound; however, it should be toned down. Currently, line 269-271 state that this CAN solve the mentioned issues, but this should be changed to being a promising option. Further, an outlook on future steps/studies would be beneficial.

**Author Response:** Thans, we have made adjustments. Line 314-315, page 13.

**Question5:**Figures – Statistical significance should be added to Figure 1. N number should either be shown in the figure or mentioned in the figure description.
Changing the format of Figure 4 would greatly improve the presentation of the results. It would be useful to add the numbers of down/up regulated metabolites to the bars, and possibly changing the y-axis to use both directions. Example: Figure 6 in Tao T, Zhao L, Lv Y, Chen J, Hu Y, Zhang T, et al. (2013) Transcriptome Sequencing and Differential Gene Expression Analysis of Delayed Gland Morphogenesis in Gossypium australe during Seed Germination.

**Author Response:** Thans, We have updated Figure 4.
