# Supplementary material for: Extraction, analysis, and antifungal activity study of algae antibiotic active substances in plateau lakes
Source: PLoS One. 2025 May 8;20(5):e0319853. doi: 10.1371/journal.pone.0319853 (PMC12061390; doi:10.1371/journal.pone.0319853)
Supplement: Attachment — Submitted filename: Response_to_reviewers_auresp_3.docx [file pone.0319853.s009.docx]

Dear Editor-in-Chief

We would like to have our revised version of manuscript entitled “**Extraction, analysis, and antifungal activity study of algae antibiotic active substances in plateau lakes”** considered for publication in **“Plos one”.**

All the comments and suggestions are addressed in the revised version and track

changes are mentioned inside the revised file for editors and reviewers. The raw data has been uploaded to the supporting Information.

**Extraction, analysis, and antifungal activity study of algae antibiotic active substances in plateau lakes**

**Reviewer 3:**

**Question1**: Line 278 rather change to 'Two principal components (PC1 and PC2) were obtained during PCA Analysis.....
Also give the relevance of this to your study

**Author Response:** PCA analysis was only used to analyze the degree of difference between each group of samples and was not very relevant to the purpose of the study. After discussion among all authors, we have decided to remove this section.

**Question2**: Indicate the column for the accurate mass and one for the MS/MS fragment ions in the UPLC MS/MS data in the suplementary.

**Author Response:** Thanks for this important suggestion. We have already indicated the column for the accurate mass and the MS/MS fragment ions.

**Reviewer 4:**

**Question1**:- 47: This should be plural ‘flavonoids’.

**Author Response:** Many thanks for this comment.We have made adjustments to the abstract section.Line 47, page 1.

**Question2**:Statistical analysis. Please add whether any outliers were excluded and if so, what method was used to determine outliers e.g. ROUT or Grubbs’ test. Line 206-207, page 8.

**Author Response:**Thanks. Outliers have been excluded in the statistical analysis, and the Tukey test was performed. We have added this information to the revised text.

**Question3**:Please add a section that describes where data is made available e.g. repository.

**Author Response:**Thanks for this important suggestion. We have uploaded the data in a file as required by the journal.

**Question4**:This section is rather short. I recommend adding a section that highlights the novelty of the study as well as a section that describes future research and use of the findings.

**Author Response:** Thanks, We have adding a section that the details on the innovation this research provides as well as key considerations for industrial-scale bactericide production to the discussion section, and have outlined the next steps for future studies.Line 285-295, page 11-12.

**Question5:** Please ensure figure legends have proper descriptions of the figure. For example, add more details to the legends of figures 4 and 5. The figure legend should provide sufficient information regarding the experiment performed and the data displayed that allows the reader to understand what is shown without having to read the entire manuscript section. Please add the p-value information and n numbers to all figures where it applies.

**Author Response:** Thans, A legend has been added to Figure 4, and specific p-values as well as the number of metabolites in each pathway have been added to Figure 5.
