# Supplementary material for: Extraction, analysis, and antifungal activity study of algae antibiotic active substances in plateau lakes
Source: PLoS One. 2025 May 8;20(5):e0319853. doi: 10.1371/journal.pone.0319853 (PMC12061390; doi:10.1371/journal.pone.0319853)
Supplement: Attachment — Submitted filename: Response_to_reviewers_auresp_4.docx [file pone.0319853.s010.docx]

Dear Editor-in-Chief

We would like to have our revised version of manuscript entitled “**Extraction, analysis, and antifungal activity study of algae antibiotic active substances in plateau lakes”** considered for publication in **“Plos one”.**

All the comments and suggestions are addressed in the revised version and track

changes are mentioned inside the revised file for editors and reviewers.

**Extraction, analysis, and antifungal activity study of algae antibiotic active substances in plateau lakes**

**Journal Requirements:**Please review your reference list to ensure that it is complete and correct. If you have cited papers that have been retracted, please include the rationale for doing so in the manuscript text, or remove these references and replace them with relevant current references. Any changes to the reference list should be mentioned in the rebuttal letter that accompanies your revised manuscript. If you need to cite a retracted article, indicate the article’s retracted status in the References list and also include a citation and full reference for the retraction notice.
**Author Response:** We have checked all the references as required and made revisions accordingly.
