# Supplementary material for: Extraction, analysis, and antifungal activity study of algae antibiotic active substances in plateau lakes
Source: PLoS One. 2025 May 8;20(5):e0319853. doi: 10.1371/journal.pone.0319853 (PMC12061390; doi:10.1371/journal.pone.0319853)
Supplement: Attachment — Submitted filename: Response_to_reviewers_auresp_5.docx [file pone.0319853.s011.docx]

Dear Editor-in-Chief

We would like to have our revised version of manuscript entitled “**Extraction, analysis, and antifungal activity study of algae antibiotic active substances in plateau lakes”** considered for publication in **“Plos one”.**

All the comments and suggestions are addressed in the revised version and track

changes are mentioned inside the revised file for editors and reviewers.

**Extraction, analysis, and antifungal activity study of algae antibiotic active substances in plateau lakes**

**Reviewer #3:**The manuscript has been improved. The authors could provide the supplimentary data and avail all documents for readers

**Author Response:** All relevant data have been submitted to the “Plos one” journal. However, no suitable database has been identified for data deposition.

**Reviewer #5:** The manuscript mainly described how to extract antifungal components from algae and analyzed their antifungal activity. Thus, I think the first thing that the author needs to convince the readers is that the natural antifungal component is better than artificial in some aspects, or the manuscript stands on an unstable base in my point of view. Also, I expect to see more discussions about the advantages and disadvantages of the extraction methods used in this manuscript compared to other methods, as well as the specialty of the algae in plateau lakes. I am also concerned about the reasonability of the antifungal activity because the only antifungal indicator is Fusarium oxysporum.

**Author Response:** The natural antifungal components derived from algae may not necessarily outperform agents. However, the preparation of phenolic acid-based fungicides using algae from high-altitude lakes addresses two critical concerns: the large-scale utilization of algae resources from these lakes and the management of plant root rot diseases. Therefore, we propose that the preparation of phenolic acid-based fungicides using high-altitude lake algae is economically viable, which has been stated in lines 289-297. In this study, four methods for preparing algal phenolic acid-based fungicides were evaluated. The critical indicators for assessing the advantages and limitations of these methods are the phenolic acid content and the antibacterial activity of the extracted compounds. A detailed analysis of these parameters is presented in Table 1. *Fusarium oxysporum* is a globally prevalent soil-borne pathogenic fungus with a broad host range. It is known to cause wilt disease in over 100 plant species, including melons, eggplants, bananas, cotton, legumes, and ornamental flowers. Our findings indicate that algal phenolic compounds exhibit significant inhibitory activity against F. oxysporum. These findings highlight their potential for large-scale application as a suitable solution for managing plant fungal diseases.

**Minor concerns:**
Line 29: Excess “the”.
**Author Response:** Many thanks for this comment. We have corrected this mistake.

Line 32: Should be “anti-plant”.
**Author Response:** Thanks, We have corrected it.

Line 210-216: The result of this part is not displayed, readers could not know where “range of 1–9 μg/ml” are from, also cannot understand why “precision and stability within 2 hours” could come from the corresponding results.

**Author Response:** Thanks for this important suggestion. We have provided a detailed method for determining the phenolic acid content

Line 253: There is only one subtitle in the chapter “metabolite identification”, is it necessary to use this subtitle?

**Author Response:** Thanks, We have removed the subtitle.

Line 286: Need to cite relevant articles or give the exact value of algae and crop yield.
**Author Response:** Thanks for this important suggestion. We have provided the cite articles, line 292-295.

Figure 1: It is recommended to use the colored figure rather than monochrome.

**Author Response:** Many thanks for this comment. Figure 1 has been modified to a color graphic.
